# From outbreak to opportunity: Sierra Leone’s mpox crisis as a wakeup call for pandemic equity

**DOI:** 10.1186/s12939-025-02725-7

**Published:** 2026-01-06

**Authors:** Alpha Umaru Bai-Sesay, Rosetta Doreen Jones, Daniel Karim Dauda Sesay

**Affiliations:** 1 Ministry of Health/National Public Health Agency, Freetown, Sierra Leone; 2 Research and Scientific Division, Sustainable Health Systems, Freetown, Sierra Leone; 3 Insights for Development Impact, Freetown, Sierra Leone

**Keywords:** Health equity, Mpox, HIV, Sierra Leone, Global health, Pandemics, Health policy

## Abstract

The 2025 clade IIb mpox outbreak in Sierra Leone, intersecting with a significant HIV burden, is not merely a health crisis but a stark manifestation of deep-seated global health inequities. This commentary argues that the response to this outbreak reveals a familiar and fatal pattern: the neglect of diseases within marginalized populations until they threaten high-income countries. Through analyzing disparities in clinical outcomes, diagnostic and therapeutic access, and containment policies, we critique the reactive, charity-based model of global health. We propose a paradigm shift towards proactive equity, centered on the establishment of a permanent infectious disease Equity Fund to ensure the rapid, equitable distribution of vaccines, therapeutics, and diagnostics for future outbreaks, transforming a moment of crisis into an opportunity for durable change.

## The convergence of two pandemics

The World Health Organization’s situation reports often reduce human suffering to statistics. The figure of over 4,800 confirmed mpox cases in Sierra Leone in the first half of 2025 is one such statistic [1, 2]. Behind it lies a more harrowing truth: the devastating synergy of mpox and advanced HIV disease. As detailed in a recent Lancet correspondence, while mpox is often self-limiting in immunocompetent individuals, it becomes a “debilitating and life-threatening” infection for those with untreated or advanced HIV [1]. In one treatment center in Freetown, a staggering 83% of admitted mpox patients were co-infected with HIV, the majority presenting with CD4 counts below 200 cells per mm³ [1]. This confluence is not coincidental; it is diagnostic of a health system strained by historical underinvestment and a global apparatus that consistently fails the most vulnerable.

Although 5% of cases occurred in children, the overwhelming burden fell on a marginalized adult population, those living with HIV who are systemically failed by fractured care systems. The authors of the Lancet correspondence note that most patients were either newly diagnosed with HIV or had interrupted treatment, a direct consequence of the worldwide budget cuts that impede consistent access to HIV services [1]. This outbreak, therefore, is not a new problem but a symptom of a chronic one: the structural inequity that determines who is protected from pandemics and who is left exposed.

## Disparities in care beyond the virus

The clinical presentation of mpox in immunocompromised patients in Sierra Leone, characterized by large, necrotic lesions and protracted courses is a biological expression of immunological despair [1]. However, the inequities extend far beyond virology into the very architecture of the response.

The therapeutic desert is glaring. As of August 2025, not a single patient in Sierra Leone had received tecovirimat or brincidofovir [1]. This is despite guidelines from the U.S. Centers for Disease Control and Prevention recommending tecovirimat for immunocompromised patients [3, 4]. This absence is not a medical oversight but a political and economic one. The PALM007 trial, which showed limited efficacy for tecovirimat in a different mpox clade (Ia) in a largely immunocompetent population, may have inadvertently stifled urgency for deployment in West Africa [5, 6]. This exemplifies a pervasive bias: interventions are often tested in one context and deemed universally ineffective, neglecting the specific needs of the most vulnerable populations for whom the benefit-risk calculus is entirely different.

Isolation protocols become a form of punishment. WHO guidelines for ending isolation, requiring all lesions to crust over and a new skin layer to form are practically unachievable for patients with advanced HIV, who may have positive PCR tests and open wounds for months [1, 7]. In high-income settings, supportive care can manage this. In low-resource settings, prolonged isolation creates catastrophic barriers, preventing access to surgery, family, and livelihood. This policy, developed without consideration for immunocompromised hosts, exacerbates stigma and suffering, highlighting how a one-size-fits-all approach is inherently inequitable.

The vaccination campaign, while commendable for vaccinating over 11,000 people with HIV, faces an evidence gap. The immunogenicity and durability of protection for those with CD4 counts less than 200 cells per mm³ remain uncertain [1, 3, 8]. This uncertainty is a luxury these patients cannot afford. The global response was characterized by a familiar sequence: delayed vaccine donations to Africa only after the threat to the Global North had subsided. This “trickle-down biosecurity” ensures that tools arrive too late to break chains of transmission, serving more as a geopolitical pacifier than an effective public health instrument.

## Lessons for the world beyond clade IIb

The crisis in Sierra Leone offers three critical lessons for global health security.

First, pathogens exploit fault lines. Mpox did not create the vulnerabilities in Sierra Leone’s health system; it exploited them. The high rates of untreated HIV, the lack of diagnostic capacity, and the scarcity of essential medicines are pre-existing conditions. A truly equitable approach invests in strengthening these systems continuously, not just when a new pathogen makes headlines.

Second, data from one context cannot be blindly applied to another. The decision-making around tecovirimat was hamstrung by data from a different mpox clade and a different patient population. Global treatment guidelines and research agendas must be inclusive from the outset, ensuring that studies are powered to assess efficacy in immunocompromised and other vulnerable groups.

Third, isolation without support is unethical. Infection prevention and control measures must be adaptable and compassionate. For patients with prolonged infectiousness, policies must include provisions for safe, supported isolation that do not compound their social or medical suffering.

Fourth, domestic political and economic contexts shape outcomes. Political and economic actions or inactions by an affected country can divert prudent medical intervention. This kind of scenario should always be anticipated and addressed within the national context through robust governance, transparent budgeting, and accountability mechanisms to ensure that global resources are effectively deployed upon arrival.

## A vision for proactive equity

Reacting to each outbreak with ad hoc donations is a failing strategy. We must build a system that embeds equity into its core. We propose the immediate establishment of an Infectious Disease Equity Fund, a model that could be replicated for other neglected threats.

This fund would be a pre-negotiated, permanent financing mechanism governed by a coalition of endemic countries, civil society organizations and global health agencies (WHO, Africa CDC). Its mandate would be threefold:

1. **Procurement and Logistics**: To maintain a rotating emergency stockpile of specific infectious disease antivirals (for e.g. For Mpox, tecovirimat, brincidofovir), vaccines, and advanced wound care kits. The fund would manage procurement at scale to reduce costs and pre-position supplies in regional hubs for rapid deployment at the first sign of an outbreak.
2. **Research and Adaptation**: To fund targeted operational research in endemic regions to answer the critical questions raised in Sierra Leone: optimal antiviral regimens for advanced HIV, duration of infectiousness, and adapted isolation guidelines. This ensures the response is driven by context-specific evidence.
3. **System Strengthening**: To provide dedicated grants to integrate mpox and other similar threatening infectious disease surveillance, diagnostics, and care into existing HIV and primary care platforms. This builds resilience by layering new response capabilities onto established systems.

Financed through mandatory contributions from G20 nations based on a percentage of their domestic infectious disease response spending, this model would be operationalized through a legally binding international agreement. Contributions could be calculated as a small, fixed percentage (e.g., 0.1–0.5%) of a nation’s annual domestic public health emergency preparedness budget, ensuring predictability and fairness. This model moves beyond charity to justice. It acknowledges that pandemic preparedness is a global public good and that those with the greatest capacity have the greatest responsibility.

## Conclusion

The patients in Freetown with necrotic lesions and shattered immune systems are the canaries in the coal mine of global health. Their suffering is a direct result of choices, choices to defund HIV programs, to neglect stockpiles for diseases of the poor, and to prioritize national security over human security.

Sierra Leone’s outbreak is an opportunity to make a different choice. It is a wake-up call to reject the cycle of panic and neglect and to finally build a global health architecture that does not wait for a crisis to cross a border before it acts. Through establishing an Infectious Disease Equity Fund, we can choose to value every life equally, ensuring that the next outbreak is met not with scarcity and disparity, but with swift, effective, and equitable care for all. Concurrently, endemic countries like Sierra Leone must demonstrate leadership by strengthening domestic political commitment, integrating pandemic preparedness into national development plans, and ring-fencing health security budgets to ensure they are prepared to receive and effectively utilize global tools and funding. We must learn the lessons of Sierra Leone, not just for mpox, but for the next pandemic, and the one after that. The goal is not just to contain an outbreak, but to build a world where such devastating outbreaks are no longer inevitable.

### Limitations

This commentary is based on the analysis of published reports and the authors’ professional insights within the Sierra Leonean health system. It does not present new primary data. The proposed Equity Fund is a conceptual model whose practical implementation would require further detailed technical, legal, and political negotiation.

## Data Availability

Data sharing is not applicable to this article as no datasets were generated or analysed during the current study.

## Abbreviations

HIV: Human Immunodeficiency Virus
WHO: World Health Organization
CDC: Centers for Disease Control and Prevention
IRIS: Immune Reconstitution Inflammatory Syndrome
